# Anti-diabetic and Antioxidant Activities of a Bornean Ethnomedicinal Plant: *In vitro* and *In silico* Investigations of *Fibraurea tinctoria* Root Fractions

**DOI:** 10.21315/tlsr2026.37.3.10

**Published:** 2026-10-15

**Authors:** Yudi Purnomo, Apon Zaenal Mustopa, Jendri Mamangkey, Herman Irawan

**Affiliations:** 1 Department of Pharmacy, Faculty of Medicine, University of Islam Malang, Malang, Indonesia; 2 Research Center for Genetic Engineering, Research Organization for Life Science and Environment, National Research and Innovation Agency (BRIN), KST Soekarno, Cibinong, Indonesia; 3 Department of Biology, Faculty of Mathematics and Natural Sciences, Universitas Negeri Makasar, Makasar, Indonesia

**Keywords:** 3-Isoxazolecarboperoxoic Acid, Antioxidant, Anti-diabetic, Ethnomedicine, Borneo, Empirical Investigations

## Abstract

*Fibraurea tinctoria*, a native ethnomedicinal plant from Borneo, has been traditionally used to treat diabetes, with preclinical studies suggesting anti-hyperglycemic activity. However, the mechanisms underlying its hypoglycemic effects and active compounds remain unclear. This study aims to evaluate the anti-diabetic potential of *F. tinctoria* root extract through *in vitro* α-glucosidase inhibition and antioxidant assays, supported by *in silico* analysis. The roots were extracted using methanol, followed by fractionation with n-hexane (Fraction C6), ethyl acetate (Fraction EtOAc), *n*-butanol (Fraction BuOH) and aqueous solvent (Fraction H_2_O). The α-glucosidase inhibition assay was conducted using p-nitrophenyl-α-D-glucopyranoside (p-NPG), and antioxidant activity was measured using the DPPH method. Data were presented as mean ± SD, with IC_50_ values calculated by linear regression. Fraction EtOAc exhibited the strongest inhibitory activity against both α-glucosidase (IC_50_ = 147 ± 3.18 μg/mL) and free radicals (IC_50_ = 155 ± 2.30 μg/mL) compared with other fractions, though lower than reference standard acarbose and ascorbic acid. Phytochemical analysis using GC-MS identified bioactive compounds, further confirmed by molecular docking. 3-Isoxazolecarboperoxoic acid demonstrated optimal interactions with α-glucosidase (3TOP) and the aldose reductase (1IEI). These findings suggest that *F. tinctoria* could be a promising source for novel anti-diabetic and antioxidant agents.

**HIGHLIGHTS**

- *Fibraurea tinctoria* root, particularly the ethyl acetate fraction, demonstrates potent anti-diabetic properties through effective α-glucosidase inhibition.
- The ethyl acetate fraction also exhibits strong antioxidant activity, indicating potential for managing oxidative stress linked to diabetes.
- 3-Isoxazolecarboperoxoic acid was identified as a key compound, showing interactions with enzymes relevant to both hyperglycemia and oxidative stress.

## INTRODUCTION

Diabetes mellitus (DM) is a group of physiological disorders characterised by hyperglycemia, resulting from abnormalities in carbohydrate, fat, and protein metabolism due to insulin resistance, insufficient insulin secretion or a combination of both (Jellinger 2007). According to the *Diabetes Atlas* 2021, approximately 537 million people are currently living with diabetes mellitus, a figure projected to rise to 643 million by 2030 and to 783 million by 2045, representing an increase of nearly 46% (Kondo 2020; Singh 2023). In 2017, diabetes was a leading cause of death globally, responsible for around 5 million deaths, with global healthcare expenditure on the disease estimated at USD850 billion (Cho *et al*. 2018). This epidemic presents significant challenges to public health, healthcare systems, and economies globally (Duarte *et al*. 2018). Diabetes is associated with a range of complications, including both micro- and macrovascular conditions (Fowler 2008). Effective management requires a multidisciplinary approach involving healthcare professionals, patients and families. To mitigate this growing crisis, innovative strategies such as predictive diagnostics and targeted prevention are essential to reversing current trends in diabetes care.

Individuals with DM typically experience elevated oxidative stress due to persistent chronic hyperglycemia, which impairs the antioxidant defense system and promotes free radical production (Scott & King 2004). Free radicals in patients with diabetes contribute to both micro- and macroangiopathy (Salem *et al*. 2015). Excessive production of reactive oxygen species (ROS) overwhelms the body’s antioxidant defenses, leading to oxidative stress that damages cell membranes and vital macromolecules, such as DNA, proteins and lipids (He *et al*. 2017). In diabetes, excess free radicals are generated through glucose oxidation and non-enzymatic protein glycation, causing cellular damage that weakens the antioxidant defense system and contributes to complications such as nephropathy, retinopathy and neuropathy (Aruoma *et al*. 2007; Luna & Estévez 2018). Recently, attention has shifted toward exploring natural antioxidants to mitigate the effects of diabetes.

Dietary antioxidants, including vitamins A, C and E, plant polyphenols, carotenoids, flavonoids and alpha-lipoic acid, play a crucial role in protecting against diabetes by preserving beta-cell function and reducing complications (Shafras *et al*. 2024). Plants rich in natural antioxidants have demonstrated anti-diabetic effects in various studies, with bioactive compounds such as phenolics and polysaccharides shown to lower blood glucose levels (Sun *et al*. 2021). Although antioxidants are increasingly recognised for their role in managing oxidative stress-related diabetic pathologies, its efficacy remain to be fully established. Yellow root (*Fibraurea tinctoria*) is traditionally used in Borneo’s indigenous medicine. The Dayak, Banjar, Melayu and Kutai tribes use water extracts from both the root and stem of *Fibraurea tinctoria* to treat hepatitis, malaria and diabetes (Noorcahyati *et al*. 2016). One of the key secondary metabolites of yellow root is berberine, an isoquinoline alkaloid known for its antimicrobial, anti-inflammatory, anti-malarial, anti-cancer and anti-diabetic properties (Wongbutdee 2009). This compound is commonly found in various medicinal plants and has been used to treat diabetes mellitus in traditional Chinese, Indian and Middle Eastern medicine. Additionally, several studies have demonstrated that berberine possesses antioxidant activity, which may contribute to its therapeutic efficacy against diabetes (Li *et al*. 2014). The administration of ethanolic extracts of *F. tinctoria* at doses of 50, 100 and 200 mg/kg body weight has been shown to significantly reduce blood glucose levels in rats induced with alloxan (Supomo *et al*. 2024). However, the precise mechanisms of its anti-diabetic and antioxidant effects, as well as its active compounds, remain incompletely understood. Therefore, the aim of this study was to evaluate the anti-diabetic potential of *F. tinctoria* root fractions through α-glucosidase inhibition and antioxidant activity with *in vitro* and *in silico* approaches.

## MATERIAL AND METHODS

### Sample Preparation

*Fibraurea tinctoria* root was obtained from Borneo Island and then pulverised and identified by the Laboratory of UPT Materia Medika Batu, Malang, Indonesia, with certificate number 074/027/101.8/2019. A 50 g portion of the powdered root was extracted with 250 mL of methanol using a shaker for 6 h. This extraction was repeated three times with fresh solvent. After evaporating the solvent, 15 g of the concentrated extract was subjected to fractionation by partitioning with n-hexane (fraction C6), ethyl acetate (fraction EtOAc), n-butanol (fraction BuOH) and water (fraction H_2_O). Each fraction was then evaporated to yield a paste-like substance. For the oral glucose tolerance test, 10 g of the simplicia was extracted using a decoction method with 250 mL of hot water at 90°C for 30 min.

### Animals and Treatments

Male Sprague-Dawley (SD) rats, weighing 180 g–200 g, were obtained from Gajah Mada University, Yogyakarta, Indonesia. All procedures followed ethical guidelines and were approved by the Commission of Ethical Research at Brawijaya University, Malang, Indonesia (certificate number 245-KEP-UB). The rats were individually housed in an automatically controlled animal room at 25 ± 1°C, with a 12:12-hour light-dark cycle. The rats were provided with standard food and water *ad libitum* and fasted overnight prior to the experiments.

### Oral Glucose Tolerance Test

Glucose was administered orally at a dose of 2 g/kg body weight following an overnight fast. *F. tinctoria* root extract at dose 250, 500 and 1000 mg/kg body weight were given 30 min before glucose induction. Blood samples were collected from the tail vein at 0 (before glucose administration), 15 min, 30 min, 60 min, 90 min and 120 min after glucose administration. Blood glucose levels were measured immediately using a commercially available glucometer. The area under the postprandial glucose curves (AUC) was calculated using the trapezoidal method.

### Anti-diabetic Bioassay

The α-glucosidase inhibitory activity of *F. tinctoria* root extract and its fractions was evaluated using p-nitrophenyl-α-D-glucopyranoside (p-NPG) as the substrate in 0.1 M potassium phosphate buffer (pH 6.8). Similarly, 0.1 unit/mL α-glucosidase was dissolved in potassium phosphate buffer. Each sample was dissolved in methanol, and 30 μL of the sample or control was added to 30 μL of α-glucosidase and 60 μL of p-NPG substrate. The mixture was incubated at 37°C for 40 min, followed by the addition of 120 μL of 0.2 M sodium carbonate in phosphate buffer (pH 6.8) to terminate the reaction. The amount of released p-nitrophenol (pNP) was measured at 405 nm, with acarbose used as the reference drug.


$$\%Inhibition=\frac{Absorbance_{Control}-Absorbance_{Sample}}{Absorbance_{Control}}\times 100\%$$

### Antioxidant Bioassay

The antioxidant potential of *F. tinctoria* root extract and its fractions was assessed using the 2,2-diphenyl-1-picrylhydrazyl (DPPH) radical scavenging method. All samples were dissolved in methanol, and 20 μL of each sample or control was added to 180 μL of 0.2 mM DPPH solution (prepared in MeOH) in a microplate. The mixture was incubated in the dark for 30 min. Ascorbic acid (50 mg/mL) was used as the reference standard. The absorbance of the solution was measured at 540 nm using a microplate reader.


$$\%Inhibition=\frac{Absorbance_{Control}-Absorbance_{Sample}}{Absorbance_{Control}}\times 100\%$$

### Identification of *F. tinctoria* Phytoconstituents By GC-MS

EtOAc fraction of *F. tinctoria* was dissolved in methanol, and 1 μL of the solution was injected into a GC-MS QP2010 Ultra (Shimadzu, Japan) system. The analysis was carried out on an Rtx®-5MS (Crossbond® 5% diphenyl/95% dimethylpolysiloxane) capillary column (30 m × 0.25 mm × 0.25 μm). The injector and detector temperatures were set at 230°C, while the oven temperature was programmed to increase from 60°C to 325°C at a rate of 15°C per min, holding at 60°C for 2 min and at 325°C for 6 min. The analysis was performed in splitless mode with a carrier gas pressure of 68.4 kPa. Identification and structural confirmation of phytoconstituents were conducted using an ISQ single quadrupole mass spectrometer (Shimadzu, Japan) operating in electron impact mode (70 eV). Mass spectra were recorded in full scan mode with a mass range of m/z 50–550 amu.

### Molecular Docking

The computational analysis was performed on an MSI operating system equipped with a 12th Gen Intel® Core™ i7-12650H processor (2.70 GHz), 16 GB RAM and an NVIDIA GeForce RTX 3050 GPU. The structures of the phytoconstituents were retrieved from the PubChem database (https://pubchem.ncbi.nlm.nih.gov) and prepared for docking by optimizing them to achieve stable geometric configurations. The macromolecular protein targets, α-glucosidase (PDB ID: 3TOP) (https://www.rcsb.org/structure/3TOP) and aldose reductase (PDB ID: 1IEI) (https://www.rcsb.org/structure/1IEI), were obtained from the Protein Data Bank (https://www.rcsb.org/). Molecular docking simulations were conducted using the PyRx 0.8 software. The docking results were then visualised and analysed with Discovery Studio Visualizer ver. 21.1.0.20298.

## RESULTS

### Anti-diabetic Effects of *F. tinctoria* Root Decoction in Rats

The blood glucose levels observed in the different treatment groups over the course of a 2-hour oral glucose tolerance test reveal varying degrees of glucose control (Fig. 1). In the control group, blood glucose levels rose from 89.3 ± 6.7 mg/dL to 147.3 ± 13.7 mg/dL at 30 min, then decreased to 104.3 ± 6.6 mg/dL by 120 min. The 250 mg/kg dose of *F. tinctoria* showed an initial glucose level of 101.5 ± 11.07 mg/dL, a modest increase to 107.5 ± 8.2 mg/dL at 30 min, and a drop to 87.0 ± 12.35 mg/dL by 120 min. The 500 mg/kg dose peaked at 114.5 ± 11.08 mg/dL before returning to 89.5 ± 12.4 mg/dL, while the 1000 mg/kg dose reduced glucose to 84.5 ± 12.1 mg/dL after peaking at 114.0 ± 1.63 mg/dL. Acarbose, the reference drug, showed the strongest effect, lowering glucose from 129.0 ± 9.09 mg/dL to 61.0 ± 11.05 mg/dL by 120 min. These findings indicate a dose-dependent effect of *F. tinctoria* on glucose regulation. Fig. 2 shows that *F. tinctoria* at doses of 250, 500 and 1,000 mg/kg body weight significantly decreased area under the curve (AUC) of glucose about 15%–16% compared with control group (*p* < 0.05). There were no significant differences between the doses of herbs and acarbose as a reference drug (*p* > 0.05). Meanwhile, acarbose exhibited the strongest decreased in AUC of glucose approximately 20% compared with control group (*p* < 0.05). These findings suggest that *F. tinctoria* has the potential to lower postprandial glucose levels, although the standard anti-diabetic drug acarbose remains higher.

### Anti-diabetic Properties of *F. tinctoria* Root Extract and Its Fractions

Table 1 and Fig. 3 highlights the anti-diabetic activity of *F. tinctoria* extracts and its fractions are measured as IC_50_ values (the concentration required to inhibit 50% of the target activity). Fraction EtOAc (IC_50_ = 155 ± 2.30 μg/mL) demonstrated the most potent activity followed by fraction H_2_O (IC_50_ = 155 ± 2.30 μg/mL), extract MeOH (IC_50_ = 308 ± 3.54 μg/mL) and fraction C6 (IC_50_ = 334 ± 20.20 μg/mL). Conversely, the fraction BuOH (IC_50_ = 705.64 ± 32.92 μg/mL) exhibited the weakest activity. The lowest IC_50_ value was observed in acarbose (IC_50_ = 14.26 ± 0.47 μg/mL), as a reference drug, with an indicating its superior efficacy in inhibiting the target enzyme.

### Antioxidant Properties of *F. tinctoria* Extract Fractions

The antioxidant activity of various *F. tinctoria* fractions compared with ascorbic acid, measured as IC_50_ values (Table 2 and Fig. 4). Fraction EtOAc (IC_50_ =147 ± 3.18 μg/mL) showed the strongest antioxidant effect followed by fraction BuOH (IC_50_ = 168 ± 1.55 μg/mL), MeOH extract (896 ± 42.81 μg/mL) and fraction C6 (IC_50_ =940 ± 67.01 μg/mL). However, fraction H_2_O demonstrated the weakest antioxidant activity, with an IC_50_ greater than 3,000 μg/mL, indicating very low-to-no antioxidant properties. Ascorbic acid (IC_50_ = 24 ± 0.56 μg/mL) as a reference standard exhibited the lowest value and indicating its potential antioxidant.

### Phytoconstituents of EtOAc Fraction of *F. tinctoria*

The GC-MS analysis of the sample (Fig. 5) revealed a variety of important chemical constituents with distinct biological and chemical properties. The most abundant compound detected was Acetic acid (Peak 1), a member of the carboxylic acid group, accounting for 60.74% of the total area. Trichloromethane (Peak 4), also known as chloroform, represented 9.88% of the sample. A haloalkane, this compound is widely recognised for its role as an organic solvent and its historical use as an anesthetic. However, due to its toxicity and potential environmental impact, its presence is noteworthy for its implications in safety and environmental discussions. Two silane derivatives were identified: Silane, dimethoxydimethyl- (Peak 5) and Silane, trimethoxydimethyl- (Peak 6), contributing 9.24% and 1.85% to the total area, respectively. These silane compounds are often used as precursors in the synthesis of silicon-based materials, with applications ranging from coatings to electronics. Other minor compounds can be seen in Table 3. Several key phytoconstituents with notable biological activities were evidenced in *F. tinctoria*. Fucoxanthin, a xanthophyll carotenoid, is known for its potent antioxidant, anti-inflammatory and anticancer properties, suggesting potential health benefits, particularly in combating oxidative stress-related diseases. (2E,4E)-N-Isobutyltetradeca-2,4-dienamide, an amide, is recognised for its analgesic, anti-inflammatory and antimicrobial effects, highlighting its potential therapeutic uses in pain relief and infection control. Additionally, 4(15)-Selinene-11,12-diol, methyl ether, a sesquiterpene, and 5-(3-Methoxyphenyl)-1,3,4-thiadiazol-2-ylamine, a thiadiazole derivative, both contribute anti-inflammatory, antimicrobial and anticancer activities, reinforcing the extract’s potential as a source of natural therapeutic agents.

### Molecular Docking of F. tinctoria Metabolites

The potential bioactive compounds from EtOAc fraction of *F. tinctoria* root were analysed for interactions with maltase-glucoamylase or α-glucosidase (PDB ID: 3TOP) as an anti-diabetic target and aldose reductase (PDB ID: 1IEI) as an antioxidant target. The docking analysis of acetic acid with the alpha-glucosidase indicated anti-diabetic potential, with a binding affinity of −3.1 kcal/mol. Similarly, its interaction with the aldose reductase showed antioxidant potential, with a binding affinity of −3.5 kcal/mol. Acetic acid formed interactions with key amino acid residues, including Asp1279 and His1584 for alpha-glucosidase, and Tyr48 and Trp20 for aldose reductase. The compound 2,2-Dimethoxybutane exhibited a binding affinity of −4.5 kcal/mol with the α-glucosidase, interacting with the Arg1510 residue, and a binding affinity of −4.2 kcal/mol with the aldose reductase, interacting with Trp111 and Trp20 residues. Among the compounds, 3-Isoxazolecarboperoxoic acid showed the strongest binding affinities, with −6.2 kcal/mol for the α-glucosidase and −8.4 kcal/mol for the aldose reductase (Table 4) approaching the binding affinity values of the reference drugs zenarestat (aldose reductase inhibitor) and acarbose (α-glucosidase inhibitor).

## DISCUSSION

Natural products are gaining increasing acceptance and use as alternative therapies, particularly for chronic conditions like diabetes mellitus. This growing trend stems from the numerous side effects and high costs associated with conventional antidiabetic drugs (Ezuruike & Prieto 2014; Lee *et al*. 2016). In many cases, natural compounds have demonstrated synergistic interactions with synthetic anti-diabetic drugs, enhancing therapeutic efficacy (Alam *et al*. 2018). Such combinations often enable lower drug dosages and/or reduced administration frequency, which helps minimise side effects while maintaining or even enhancing the desired outcomes. Ongoing research continues to explore the potential of natural products as anti-diabetic agents, particularly to identify bioactive compounds that could serve as scaffolds for developing new therapeutic drugs (Pang *et al*. 2015). A key area of interest is the link between diabetes mellitus and oxidative stress, a well-established contributor to the disease’s progression (Ighodaro 2018; Oguntibeju 2019). The excessive production of reactive oxygen species (ROS) overwhelms the body’s enzymatic and non-enzymatic antioxidant defenses, leading to cellular damage. Addressing both hyperglycemia and oxidative stress through dual-acting agents could offer a more comprehensive approach to diabetes management.

*Fibraurea tinctoria* commonly known as yellow root (*akar kuning*) in the Bornean region (Kalimantan), Indonesia, belongs to the Menispermaceae family. This plant has been traditionally used to treat a variety of ailments, including headaches, diarrhea, diabetes, dysentery, malaria and lumbago. It is also used to enhance stamina and as an antioxidant. The plant contains numerous secondary metabolites, such as berberine, alkaloids, terpenoids, palmitin and fibleucin. Among these, berberine is a well-known isoquinoline alkaloid with a wide range of biochemical and pharmacological activities, including anti-diarrheal and anti-cancer properties (Zalizar *et al*. 2019). Based on the wide-ranging health benefits of *F. tinctoria*, this study focused on extracting bioactive compounds from its root, which were hypothesised to possess dual biological activities as both antidiabetic and antioxidant agents. Previous research has not thoroughly explored the variation in solvent extractions to isolate these potential compounds. Therefore, we employed a series of solvent extractions using n-hexane, ethyl acetate, n-butanol and aqueous solvents to maximise the identification of bioactive fractions. In this study, we evaluated the antidiabetic and antioxidant effects of *F. tinctoria* root extracts and their bioactive fractions. The supernatant fractions were further analysed using an *in silico* approach to predict and validate the biological activities of the selected compounds.

We demonstrated the antidiabetic potential of *F. tinctoria* root extract through an *in vivo* study using oral glucose tolerance test on experimental rats. Although the *in vivo* test used an aqueous decoction, while the EtOAc fraction showed the strongest *in vitro* activity, both approaches served different purposes. The decoction was used to reflect the traditional preparation of *F. tinctoria* roots, whereas fractionation was performed to identify the most active fraction. The EtOAc fraction was not tested *in vivo* because safety evaluation, toxicity assessment, and dose optimisation are needed before administering a concentrated organic solvent-derived fraction to animals. At a dose of 250 mg/kg, the aqueous extract effectively reduced blood glucose levels to 87.0 ± 12.35 mg/dL after 120 min of treatment, making this the recommended optimal dose for glycemic control. This preliminary simulation confirmed that *F. tinctoria* root extract significantly mitigates blood glucose spikes, indicating its potential as an antidiabetic agent. Previous studies, including Zalizar *et al*. (2019), have reported that *F. tinctoria* contains various secondary metabolites, including berberine, alkaloids, terpenoids, palmitin and fibleucin. Supporting this, other reports on *Berberis aristata* root extract have shown that berberine plays a key role in regulating glucose homeostasis by reducing gluconeogenesis, oxidative stress and exhibiting potent antihyperglycemic activity (Potdar *et al*. 2012; Rathi *et al*. 2013). Berberine is identified as the primary antidiabetic compound in *F. tinctoria*. According to Yin *et al*. (2008), berberine, administered at 0.5 g thrice daily, demonstrated comparable efficacy to metformin in managing type 2 diabetes, significantly reducing fasting blood glucose, HbA1c, postprandial glucose, insulin and basal insulin levels. Berberine’s antidiabetic mechanisms are multifaceted, involving insulin-mimetic activity, AMPK (5′adenosine monophosphate-activated protein kinase) activation and reducing insulin resistance through protein kinase C-dependent insulin receptor expression. Additionally, it enhances glycolysis, increases GLP-1 (Glucagon-like peptide-1) secretion, regulates its release, and inhibits DPP-IV (Dipeptidyl peptidase-4) (Potdar *et al*. 2012).

The *in vitro* antidiabetic and antioxidant tests on *F. tinctoria* root extract fractions revealed that the EtOAc fraction exhibited the most potent antidiabetic activity (IC_50_ = 155 μg/mL) and significant antioxidant activity (IC_50_ = 147 μg/mL), both indicating moderate efficacy. This study represents the first report utilising the EtOAc fraction to isolate antidiabetic and antioxidant compounds from *F. tinctoria* root extract. Previous studies on EtOAc fractions from other plant species provide supporting evidence for these findings. For instance, EtOAc fractions from *Tectona grandis* crude extract demonstrated strong antidiabetic activity by inhibiting α-glucosidase, thus delaying glucose absorption in the small intestine lumen (Sanni *et al*. 2024). Similarly, Sapkota *et al*. (2022) reported that EtOAc extract of *Bergenia ciliata* showed significant inhibitory activity against both α-amylase and α-glucosidase, making it a promising candidate for diabetes management.

The screening EtOAc fraction of *F. tinctoria* root for potential phytochemicals was profiled qualitatively and the results are summarised in Table 3. A total of 21 compounds were successfully profiled, with four key compounds, i.e. acetic acid, 2,2-dimethoxybutane, 3-isoxazolecarboperoxoic acid, and trichloromethane, were selected for further analysis using *in silico* approaches (Table 4). Acetic acid was detected as the most dominant compound in the chemical profile of the extract. A similar finding was reported in *Gymnema inodorum*, in which acetic acid was identified as a major constituent and was suggested to contribute to antimicrobial activity against *Enterococcus faecium* (Ounjaijean *et al*. 2024). The high abundance of acetic acid in plant extracts may arise from the degradation of plant volatiles or plant-derived esters (George *et al*. 2016). In addition, acetic acid, together with acetate, is involved in plant carbon metabolism and may function as a metabolic regulator (Rahman *et al*. 2024).

A molecular docking study of EtOAc fraction *F. tinctoria* root demonstrated inhibitory activity both of α-glucosidase (3TOP) and aldose reductase (1IEI) as an enzyme target, as well as an antioxidant. The *in silico* analysis identified 3-isoxazolecarboperoxoic acid as the most promising compound from the EtOAc fraction, with favourable binding affinity values for both 3TOP and 1IEI enzymes. The computational analysis predicted that 3-isoxazolecarboperoxoic acid may exhibit stronger biological activity due to its high binding affinity, indicating effective interaction with both ligand and molecular targets. This compound showed similar interactions and potency to acarbose (a reference α-glucosidase inhibitor) (ΔG: −6.2 kcal/mol),) and zenarestat (a reference aldose reductase inhibitor) (ΔG: −8.4 kcal/mol). Saad *et al*. (2026) also tested some identified compounds in the EtOAc extract of *Beilschmiedia lumutensis* using human intestinal maltase-glucoamylase or 3TOP enzyme which showed favourable ligand–enzyme interactions involving hydrogen bonding and hydrophobic contacts. The analysis revealed that the compound 3-isoxazolecarboperoxoic acid, 4,5-dihydro-5-phenyl-, 1,1-dimethyl ethyl ester interacts with the residues Asp1420(A), Arg1510(A), Ile1280(A), Trp1355(A), Phe1559(A), His1584(A), Tyr1251(A), Asp1157(A) and Met1421(A) in 3TOP, while interacting with Trp111(A), Cys298(A), Trp20(A), Phe122(A), Leu300(A) and Cys303(A) in 1IEI (Fig. 6). Furthermore, 3-isoxazolecarboperoxoic acid has previously been reported in the leaves and flowers of *Tetraena simplex* (El-Zohri *et al*. 2024), but our study claimed its first identification in the roots of *F. tinctoria*. Nevertheless, molecular docking should be interpreted as a predictive approach rather than direct evidence of biological efficacy which required experimental validation (Widiastuti *et al*. 2026).

There is still limited information regarding the medicinal aspect of 3-isoxazolecarboperoxoic acid. However, some studies have reported its analogues, including isoxazole derivatives which are well known for their broad spectrum of pharmacological activities, including analgesic, anti-inflammatory, antimicrobial, antioxidant and anticancer properties (Ruthu *et al*. 2011). Extensive research has been conducted on the synthesis and biological evaluation of novel isoxazole compounds for their therapeutic potential. For instance, Bibi *et al*. (2019) synthesised 3-substituted-isoxazole-4-carboxamide derivatives and reported moderate analgesic activity, with compounds A3 and B2 showing promising efficacy via non-opioid receptor pathways. Notably, isoxazole rings are key structural components in various drugs, including COX-2 inhibitors, β-lactamase-resistant antibiotics and synthetic androgenic steroids (Chikkula & Raja 2017). The wide-ranging pharmacological activities of isoxazole derivatives highlight their significance as valuable molecular scaffolds for drug discovery and development across diverse therapeutic areas. In addition, these findings highlight the potential of the local *F. tinctoria* plant from Borneo, Kalimantan, as a source of bioactive compounds with strong antidiabetic and antioxidant potential through appropriate extraction techniques.

## CONCLUSION

The findings of this study indicate that the fraction of *Fibraurea tinctoria* root, a Borneo ethnomedicine, holds significant potential as an alternative source of anti-diabetic and antioxidant agents, particularly from its EtOAc fraction. Thus, *F. tinctoria* root could serve as a promising natural source of bioactive compounds beneficial for human health, especially in the management of diabetes. Although our *in vivo*, *in vitro* and *in silico* studies suggest the potential of *F. tinctoria* root for antioxidants and antidiabetic applications, further in-depth *in vivo* experimentation is essential to validate and confirm these preliminary findings, ultimately supporting its therapeutic benefits.

## Figures and Tables

**FIGURE 1:**
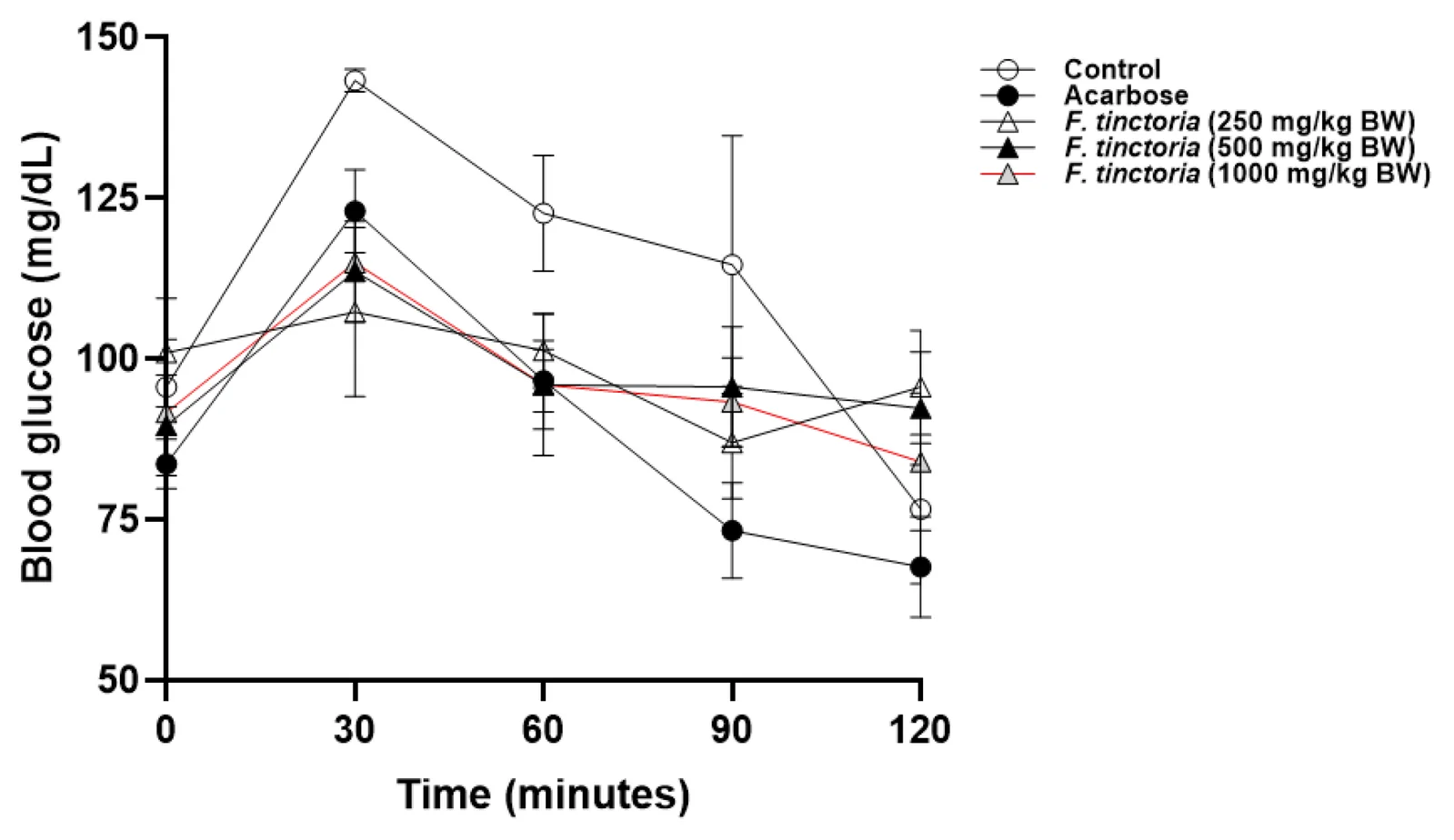
Blood glucose level in the control, acarbose and *F. tinctoria* root decoction in different doses after glucose induction on administered rats (*n* = 4).

**FIGURE 2:**
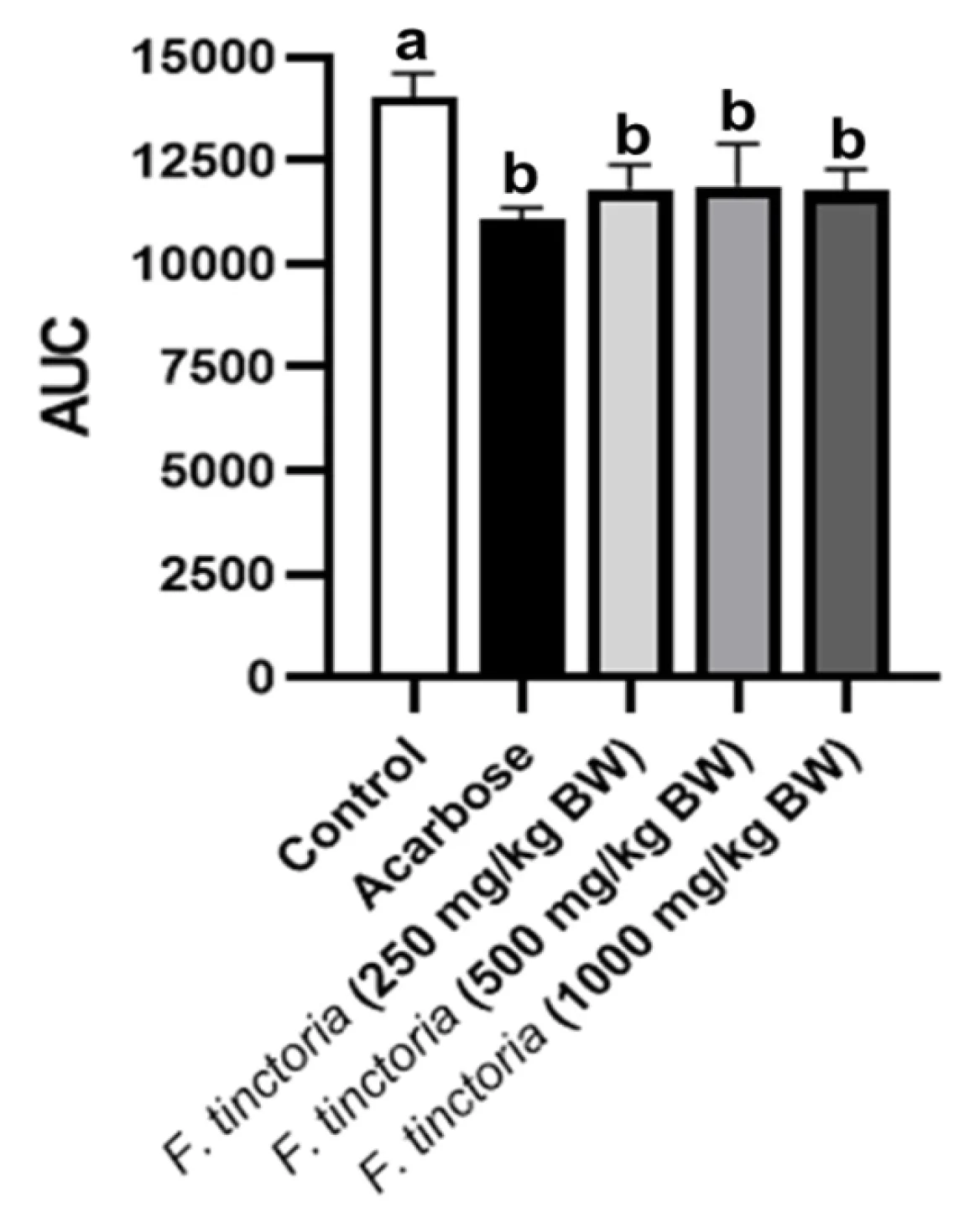
Area under curve (AUC) of blood glucose level in the control and treated rats (*n* = 4). *Note:* a, b = the different letter indicated the different effect (*p* < 0.05, LSD test).

**FIGURE 3:**
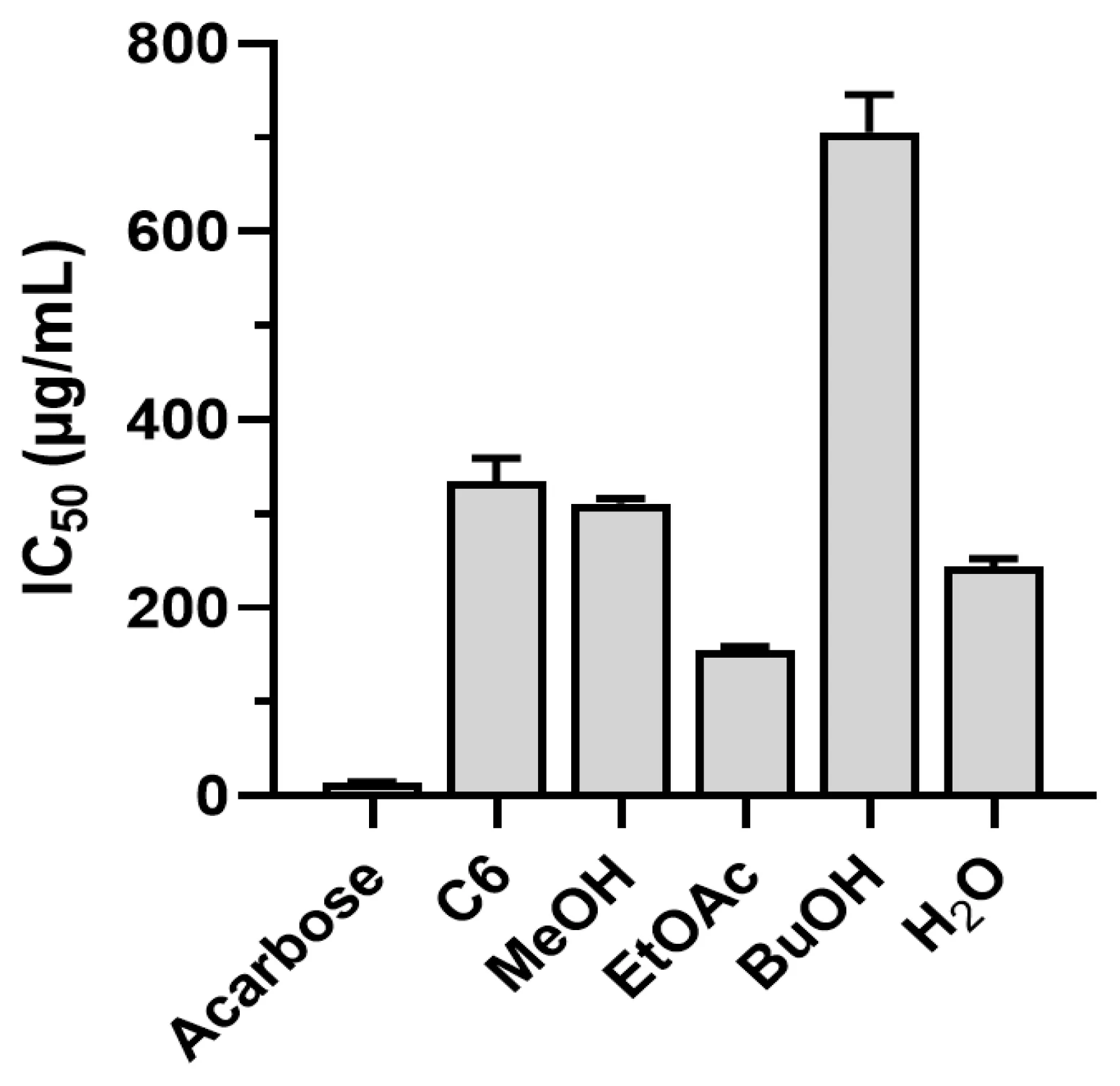
*In-vitro* anti-diabetic activities (IC_50_) of *F. tinctoria* fractions and acarbose as a control. MeOH = methanol, C6 = *n*-hexane, EtOAc = ethyl acetate, BuOH = *n*-butanol, H_2_O = water.

**FIGURE 4:**
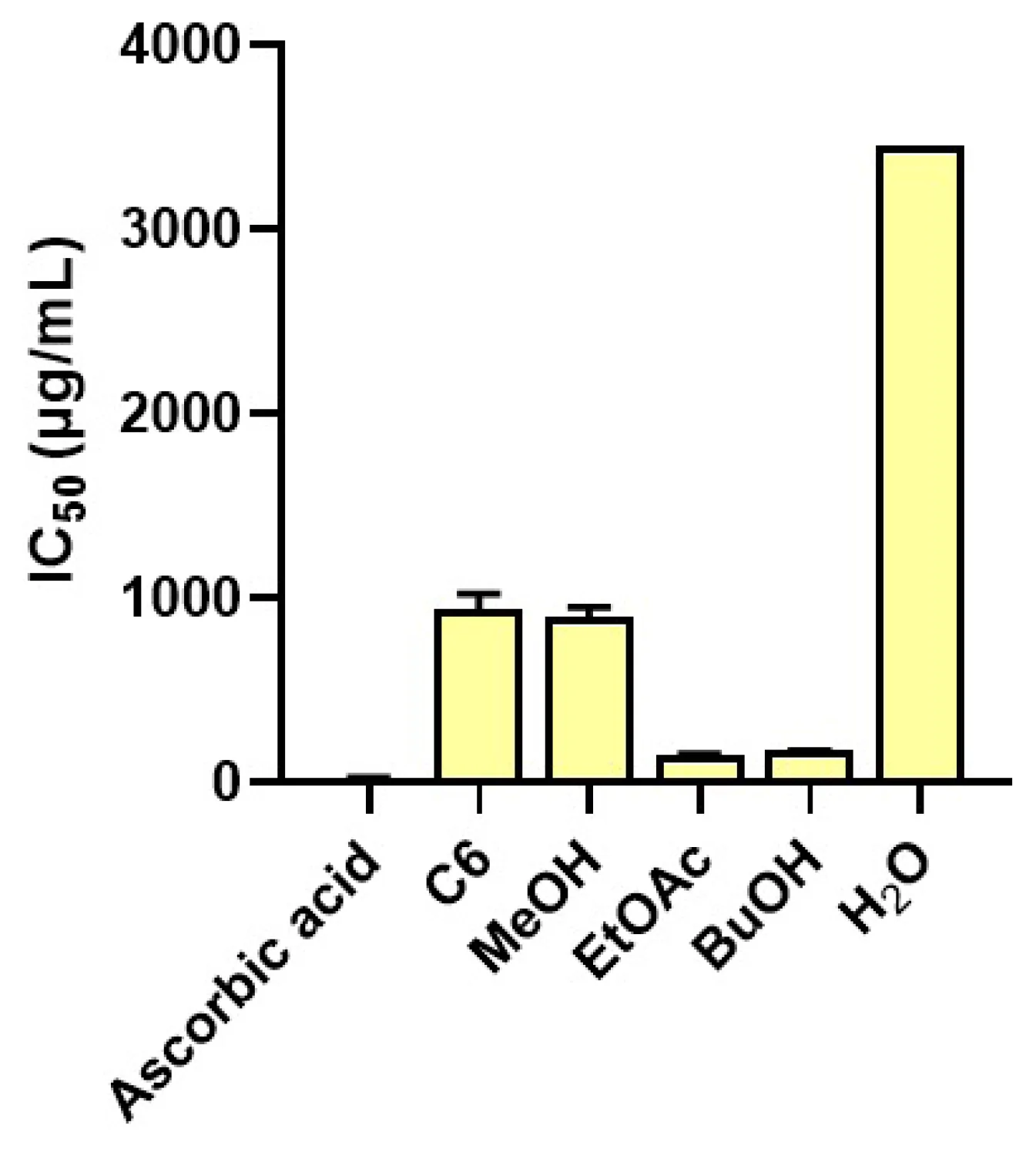
*In-vitro* antioxidant activities (IC_50_) of *F. tinctoria* fractions and ascorbic acid as a control. MeOH = methanol, C6 = *n*-hexane, EtOAc = ethyl acetate, BuOH = *n*-butanol, H_2_O = water.

**FIGURE 5:**
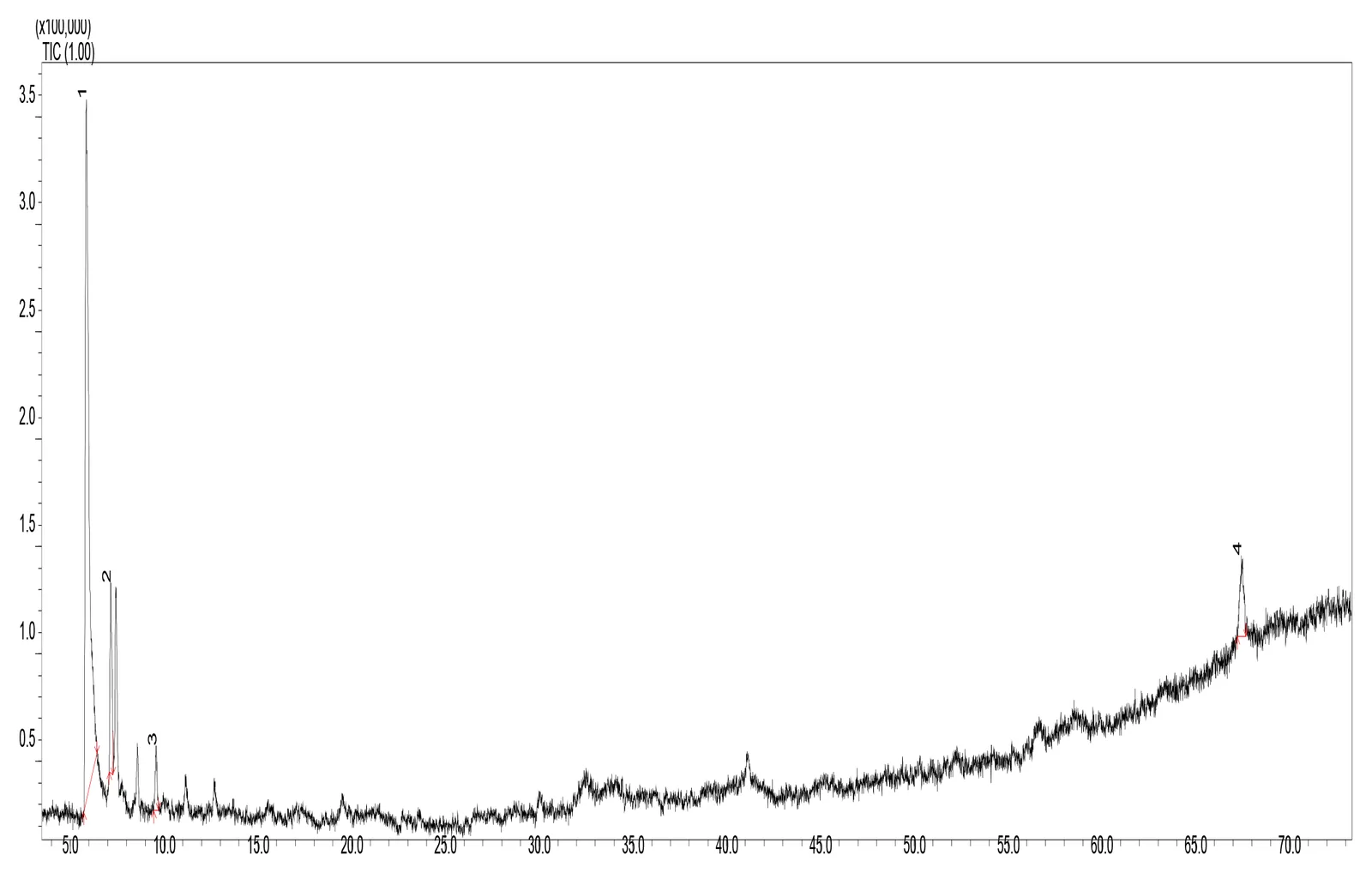
Chromatogram of EtOAc fraction of *F. tinctoria* root.

**FIGURE 6:**
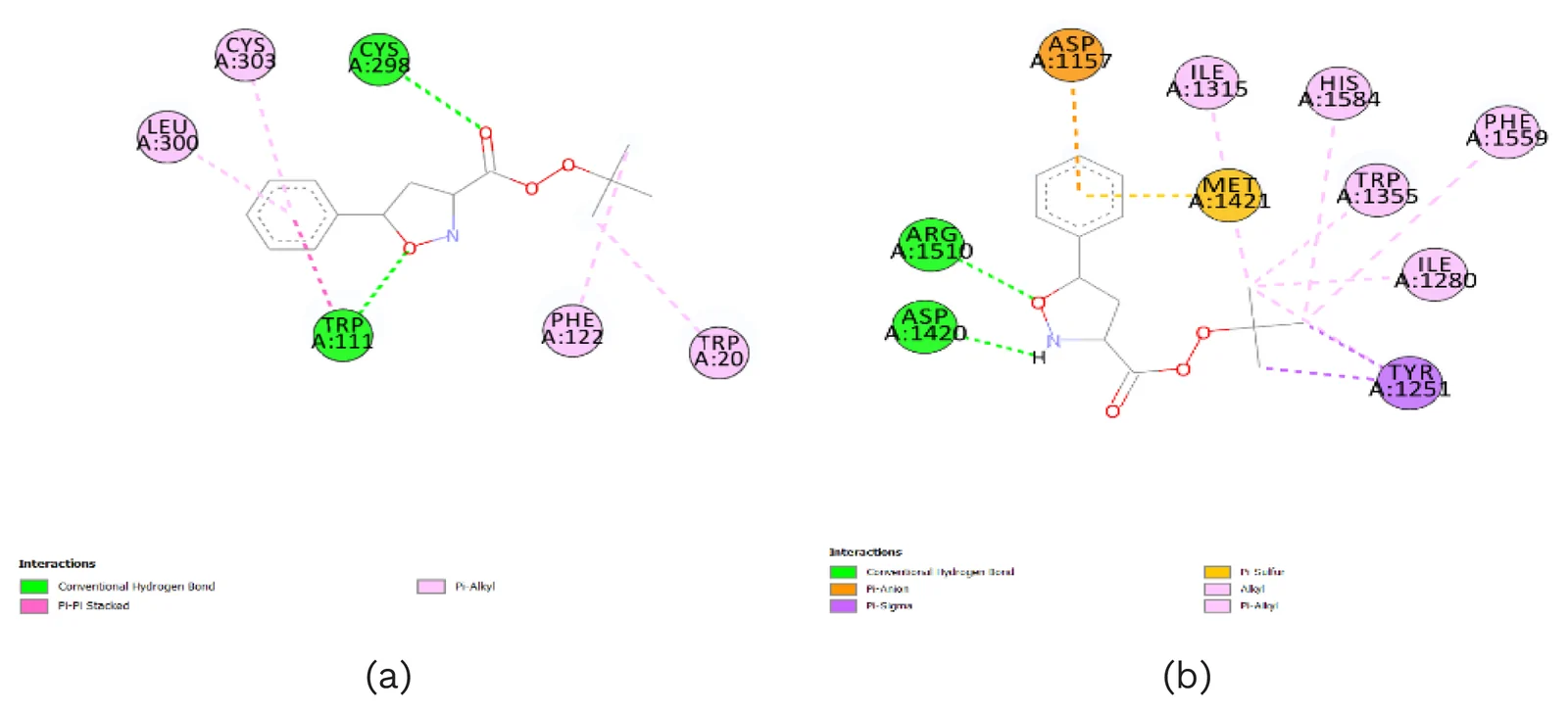
2D visualisation of ligand interaction analysis. (a) 3-isoxazolecarboperoxoic acid with 3TOP and (b) 3-isoxazolecarboperoxoic acid with 1IEI.

**TABLE 1:** Inhibition percentage of *F. tinctoria* root fractions on alpha-glucosidase and acarbose as a reference standard.

Sample (*n* = 3)	Concentration (μg/mL)	Inhibition (%)	IC_50_ (μg/mL)
Acarbose	6.25	15.52 ± 3.92	14.46 ± 0.47
	12.5	50.35 ± 1.51	
	25.0	87.43 ± 0.45	
C6 (Fraction)	50	12.10 ± 2.68	334.34 ± 20.20
	100	34.75 ± 7.93	
	200	57.95 ± 1.16	
MeOH (Extract)	100	4.17 ± 0.28	308.78 ± 3.54
	200	21.06 ± 2.41	
	400	71.39 ± 0.77	
EtOAc (Fraction)	50	11.08 ± 1.67	155.94 ± 2.30
	100	15.26 ± 1.41	
	200	72.57 ± 0.78	
BuOH (Fraction)	100	21.06 ± 2.41	705.64 ± 32.92
	200	28.01 ± 0.68	
	400	35.43 ± 1.97	
H_2_O (Fraction)	100	20.87 ± 2.41	243.38 ± 7.06
	200	35.43 ± 1.97	
	400	86.43 ± 0.58	

*Notes*: MeOH = methanol, C6 = *n*-hexane, EtOAc = ethyl acetate, BuOH = *n*-butanol, H_2_O = water.

**TABLE 2:** Inhibition percentage of *F. tinctoria* root fractions against free radicals using the DPPH assay, with ascorbic acid as the reference standard.

Sample (*n* = 3)	Concentration (μg/mL)	Inhibition (%)	IC_50_ (μg/mL)
Ascorbic acid	20	46.13 ± 1.42	23.80 ± 0.55
	40	61.38 ± 2.15	
	60	86.14 ± 0.89	
C6 (Fraction)	100	28.81 ± 0.17	334.34 ± 20.20
	200	31.12 ± 0.22	
	400	36.41 ± 0.64	
MeOH (Extract)	100	28.81 ± 0.17	898.56 ± 42.81
	200	31.12 ± 0.26	
	400	36.41 ± 0.59	
EtOAc (Fraction)	50	32.17 ± 0.08	147.61 ± 3.18
	100	40.69 ± 0.18	
	200	59.98 ± 1.11	
BuOH (Fraction)	50	32.13 ± 0.54	168.70 ± 1.55
	100	39.25 ± 0.07	
	200	55.03 ± 0.39	
H_2_O (Fraction)	100	24.00 ± 0.05	3446.89 ± 187.97
	200	24.53 ± 0.09	
	400	25.97 ± 0.45	

*Notes*: MeOH = methanol; C6 = *n*-hexane; EtOAc = ethyl acetate; BuOH = *n*-butanol; H_2_O = water.

**TABLE 3:** GC-MS profile of EtOAc fraction of *F. tinctoria* roots.

Peak	R. time	Area (%)	Compound(s)	Chemical formula	Chemical group	MW (g/mol)
1.	5.868	60.74	Acetic acid	C_2_H_4_O_2_	Carboxylic acid	60.05
2.	6.175	4.16	3-Isoxazolecarboperoxoic acid	C_14_H_17_NO_4_	Peroxy acid	263.29
3.	6.260	2.37	Dihydroniloticin	C_30_H_50_O_3_	Diterpenoid	458.7
4.	7.162	9.88	Trichloromethane	CHCl_3_	Haloalkane	119.37
5.	7.430	9.24	Silane, dimethoxydimethyl-	C_4_H_12_O_2_Si	Silane	120.22
6.	8.580	1.85	Silane, trimethoxydimethyl-	C_4_H_12_O_3_Si	Silane	136.22
7.	9.582	0.91	2,2-Dimethoxybutane	C_6_H_14_O_2_	Ether	118.17
8.	52.571	0.68	1-Bromomethylenedecahydronapthalene	C_11_H_17_Br	Alkene	229.16
9.	57.610	0.62	Isoquinoline-1-carbonitrile, 2-hex-4-ynoyl-1,2-dihydro-	C_16_H_14_N_2_O	Nitrile	250.29
10.	60.924	0.68	Benzenesolfonic acid	C_13_H_10_C_l3_NO_3_S	Sulfonic acid	366.6
11.	66.695	0.77	2-Azatricyclo[4.3.1.1(4.8)]undecane	C_10_H_17_N	Azatricyclic compound	151.25
11.	68.424	0.84	3,6,9,12-Tetraoxatetradecan-1-ol	C_10_H_22_O_5_	Polyether	222.28
12.	69.260	0.81	3,6-Methanonaphth[2,3-b]oxirene-2,7-dione, 4-bromooctahydro-5-hydroxy-	C_11_H_11_BrO_4_	Lactone	287.11
13.	69.514	0.60	4-Bromo-2-chloro-6-nitrophenol	C_6_H_3_BrClNO_3_	Phenol	252.45
Peak	R. time	Area (%)	Compound(s)	Chemical formula	Chemical group	MW (g/mol)
14.	69.849	0.67	4-(Hydroxymethyl)-6-[(thiophen-2-yl) carbonyl]-3-oxabicyclo[3.1.0] hexan-2-one	C_11_H_10_O_4_S	Bicyclic compound	238.26
15.	70.814	0.62	Fucoxanthin	C_42_H_58_O_6_	Carotenoid	658.9
16.	71.915	0.78	(2E,4E)-N-Isobutyltetradeca-2,4-dienamide	C_18_H_33_NO	Amide	279.5
17.	71.995	0.66	3,6,9,12-Tetraoxatetradecan-1-ol	C_10_H_22_O_5_	Polyether	222.28
18.	72.563	0.71	4(15)-Selinene-11,12-diol, methyl ether	C_15_H_26_O_2_	Ether	238.37
19.	72.641	1.46	5-(3-Methoxyphenyl)-1,3,4-thiadiazol-2-ylamine	C_9_H_9_N_3_OS	Thiadiazole	207.25
20.	73.041	0.94	Piperazine	C_4_H_10_N_2_	Piperazine	86.14
21.		100.00				

**TABLE 4:** Molecular docking analysis of selected compounds in the EtOAc fraction of *F. tinctoria* roots.

Chemical name	3TOP α-glucosidase binding affinity (kcal/mol)	Interacting amino acid residues in 3TOP	1IEI aldose reductase binding affinity (kcal/mol)	Interacting amino acid residues in 1IEI
Acetic acid	−3.1	Asp1279, His1584 (hydrogen bond)	−3.5	Tyr48 (hydrogen bond); Trp20 (π-stacked interaction)
2,2-Dimethoxybutane	−4.5	Arg1510	−4.2	Trp111 (hydrogen bond); Trp20 (π-stacked interaction)
3-Isoxazolecarboperoxoic acid	−6.2	Arg1510 (hydrogen bond); Ile1280, Ile1315, Met1421, His1584 (alkyl interactions); Tyr1251, Trp1355 (π-sigma interactions); Phe1427 (π-stacked interaction)	−8.4	Trp111, Cys298 (hydrogen bonds); Trp20, Tyr48, His110, Tyr209, Leu300, Cys303 (alkyl interactions)
Trichloromethane	−3.1	Not detected	−3.3	Not detected
Zenarestat, reference aldose reductase inhibitor	Not applicable	Not applicable	−9.2	Tyr48, His110, Trp111, Cys289 (hydrogen bonds); Val47, Ala299 (halogen interactions); Leu300, Cys303, Tyr309 (alkyl interactions); Trp20 (π-stacked interaction)
Acarbose, reference α-glucosidase inhibitor	−8.9	Asp1157, Asp1279, Met1421, Arg1510, Asp1526, His1584 (hydrogen bonds); Asp1420 (carbon-hydrogen bond); Tyr1251, Phe1559, Phe1560 (alkyl interactions)	Not applicable	Not applicable
